# Intimate Partner Violence and Population Mental Health: Why Poverty and Gender Inequities Matter

**DOI:** 10.1371/journal.pmed.1001440

**Published:** 2013-05-07

**Authors:** Alexander C. Tsai

**Affiliations:** 1 Center for Global Health, Massachusetts General Hospital, Boston, Massachusetts, United States of America; 2 Department of Psychiatry, Massachusetts General Hospital, Boston, Massachusetts, United States of America; 3 Harvard Medical School, Boston, Massachusetts, United States of America

## Abstract

Alexander Tsai discusses a new research study by Karen Devries and colleagues, and comments on the possible impact on public health of the study's insights regarding the relationship between intimate partner violence and mental health.

*Please see later in the article for the Editors' Summary*

**Linked Research Article**

This Perspective discusses the following new study published in *PLOS Medicine*:

Devries KM, Mak J, Bacchus L, Child J, Falder G, et al. (2013) Intimate Partner Violence and Incident Depressive Symptoms and Suicide Attempts: A Systematic Review of Longitudinal Studies. PLoS Med 10(5): e1001439. doi:10.1371/journal.pmed.1001439

Karen Devries and colleagues conduct a systematic review of longitudinal studies to evaluate the direction of association between symptoms of depression and intimate partner violence.

Steven Pinker [1] suggests that the world is becoming less violent. Yet, although it has been nearly two decades since the United Nations issued its *Declaration on the Elimination of Violence against Women*, the global burden of violence against women still remains alarmingly high [2]. Violence commonly results in physical injury, and other serious physical or psychological sequelae may also result, compounding its contribution to the overall global burden of disease [3]. The systematic review and meta-analysis by Karen Devries and colleagues [4] in this week's *PLOS Medicine* brings us one step closer toward understanding the mental health liabilities associated with violence against women. The public health impact of their contribution is substantial, given the lack of resources devoted to the prevention and treatment of mental disorders worldwide [5],[6],[7].

Their article is not the first to summarize the impacts of various forms of violence and abuse against women on mental health [8],[9],[10] (Table 1). Karen Devries and colleagues [4] contribute to this body of literature reviews by restricting their focus to longitudinal studies, thereby examining the temporal relationship between intimate partner violence and depression. Although the literature contained few studies examining the impacts of violence on incident depressive disorders, the authors identified enough studies to support a conclusion that violence was associated with incident symptoms of depression. They had intended to also examine the association between victimization and depression among men, but lack of data limited their ability to draw firm conclusions.

**Table 1:** Peer-reviewed journal articles summarizing the literature on intimate partner violence and mental health.

Author	Sample	Design	Exposure	Outcome	Findings
Golding [8]	Women^a^	36 cross-sectional studies	Physical violence by men against women	Depression^b^, suicidality, post-traumatic stress, substance use	Greater prevalence of outcomes among victims of violence
Beydoun and colleagues [9]	Women	32 cross-sectional and 5 longitudinal studies	IPV^c^	Any author definition of depression	Pooled RRs of outcomes ranged from 1.43–1.81, depending on study design and outcome; pooled RR for major depressive disorder was 2.70
Trevillion and colleagues [10]	Women^a^ or men^d^	38 cross-sectional and 3 longitudinal studies^e^	Lifetime or past-year violence or abuse by any intimate contact^c^	Mental disorders as assessed with diagnostic instruments	Pooled ORs for depressive, anxiety, and post-traumatic stress disorders among women ranged from 2.29 to 7.34; insufficient data to estimate pooled ORs for longitudinal studies
Devries and colleagues [4]	Women^a^ or men^d^	16 longitudinal studies	IPV^c^	Any author definition of depression or suicide attempts	Pooled OR for incident depression among women was 1.97 (six studies), while pooled OR for incident IPV among women was 1.93 (four studies)

a Excluded studies that assessed outcomes among women solely during the perinatal period.

b Term used (both here and throughout the article) to refer broadly to either formal diagnoses of depressive disorders or elevated symptoms of depression.

c Included psychological abuse or emotional violence.

d Insufficient data to calculate pooled estimates among men.

e Explicitly specified absence of a language restriction.

IPV, intimate partner violence; OR, odds ratio; RR, relative risk.

Notably, Karen Devries and colleagues [4] also found that, among women, symptoms of depression were associated with incident experiences of violence. Whether the relationship between violence and depression is truly causal in both directions is unknown. For example, in examining the association between exposure to intimate partner violence during adulthood and incident symptoms of depression, an unmeasured confounder preceding both variables such as childhood abuse—not measured in most of the studies—could be causally related to both irrespective of their temporal relationship with each other, inducing a spurious association that could be mistakenly interpreted as bidirectional. Statistical adjustment for baseline depression symptom severity, which was done in most of the studies, could potentially help with this problem if it is a key pathway through which the confounding effects of childhood abuse are transmitted.

So what do these findings suggest for clinical practice, programming, or policies to prevent violence against women? If depression is causally related to violence, one might consider evidence-based collaborative care management of depression [11],[12] in order to prevent subsequent experiences of violence. If violence is causally related to depression, one might suggest counseling interventions for women with histories of partner abuse in order to prevent subsequent episodes of depression, but high-quality randomized controlled trials have shown mixed results [13],[14],[15],[16]. The only randomized trial of universal screening for intimate partner violence yielded null findings [17]. It is possible that screening may exert substantive benefits only in settings where providers also have the ability to refer their clients to a broad array of services (e.g., case management, crisis services, legal advocacy, emergency shelters, transitional housing, and/or parenting and childcare support) [18], but the effectiveness of such a multipronged approach is as of yet unknown.

This particular gap in the literature redirects our attention to more systemic determinants of the excess burden of violence and depression among women. The simplified conceptual model depicted in Figure 1, informed in part by previously published work [19],[20], suggests not only that violence against women is a consequence of gendered norms about the use violence against intimate partners and power relations [21],[22], but also that its direct and indirect effects serve to reproduce these norms and power relations 23,24,25. The extent to which exposure to violence results in poor mental health outcomes is modified by gendered norms governing how men and women safely negotiate situations of potential danger [26],[27] as well as by biological differences in physical strength. And finally, poverty offers a hospitable environment for gender-unequal norms [28], violence against women [22],[29],[30], and psychological distress [31],[32] to thrive.

**Figure 1:**
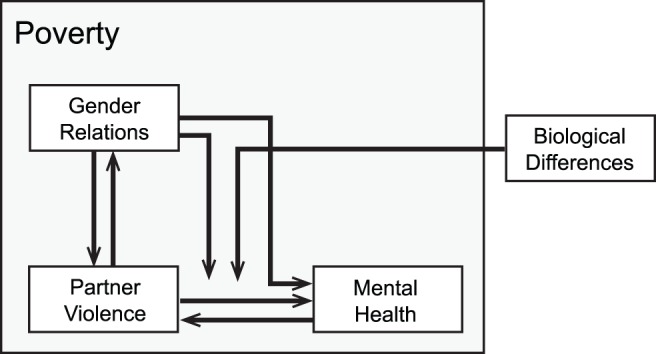
Conceptual framework depicting connections between intimate partner violence and mental health, in the context of poverty, gender relations, and biological differences between men and women.

A reduced form of this model might not be obtainable, but at the very least this model suggests that: (a) violence against women may have direct adverse effects on mental health, as suggested by Karen Devries and colleagues [4]; and, simultaneously, (b) both violence and depression are partly rooted in gender-unequal relations and the unremitting strain of poverty. Meeting this formidable challenge will require complex interventions operating at multiple levels [33],[34]. As the epidemiologist Geoffrey Rose succinctly stated in his now-classic work, “Mass diseases and mass exposures require mass remedies” [35] (p.129). Rose argued that interventions aimed at shifting the entire distribution of disease (rather than interventions aimed at preventing disease in “high-risk” individuals) would yield substantive benefits for population health even if they carry little benefit for a given individual [36],[37]. This approach emphasizes the societal changes needed to curtail the production and unequal distribution of violence against women.

With regard to the conceptual model depicted in Figure 1, effective structural interventions would target the conditions that shape women's risks for these co-occurring problems. For example, in one randomized controlled trial conducted in rural South Africa, partnered women who took part in a microfinance intervention experienced a statistically significant decline in intimate partner violence [38]. While the intervention might be considered an “agentic” type of structural intervention that targets individual behavior change [39],[40], it is plausible that participation led to environmental changes on a micro scale, such as improvements in women's bargaining power vis-à-vis their intimate partners. Thus the findings of that study [38] hint at the possibilities for change on a macro scale. Consistent with their findings, an analysis of macroeconomic data from the US showed that changes in labor market conditions favoring women led to reductions in domestic violence [41]. Related work in India showed that a policy change increasing the number of women in political leadership positions had the effect of weakening stereotypes about gender roles [42] and raising parental aspirations for their daughters' educational attainment [43].

It would be imprudent to suggest that Rose's “high-risk” strategy [37] has no place in violence prevention. The review by Karen Devries and colleagues [4] reveals major gaps in research on intimate partner violence and depression, including lack of adjustment for childhood sexual abuse or other trauma. A life course perspective, which is missing from much of the research on determinants of violence in general [44],[45], would greatly enrich the field by helping intervention programs better address histories of child abuse and/or family violence in identifying targets for secondary prevention. As we continue to advance the science of developing and testing structural interventions, we should remain vigilant for potential synergies to emerge. Quite likely, the most effective approaches will involve interventions targeting high-risk individuals, complemented with population-based approaches focused on shifting the entire frequency distribution. At this time, the effectiveness of such a combination approach may be speculative. What is clear is that we cannot sit idly by while awaiting Pinker [1]'s long arc of history to avert the persistent psychological scars of violence.
